# Interventions to mitigate COVID-19 misinformation: protocol for a scoping review

**DOI:** 10.1186/s13643-022-01917-4

**Published:** 2022-05-30

**Authors:** Navin Kumar, Nathan Walter, Kate Nyhan, Kaveh Khoshnood, Joseph D. Tucker, Chris T. Bauch, Qinglan Ding, S. Mo Jones-Jang, Munmun De Choudhury, Jason L. Schwartz, Orestis Papakyriakopoulos, Laura Forastiere

**Affiliations:** 1 Department of Biostatistics, Yale School of Public Health, New Haven, CT USA; 2 Northwestern University, Evanston, IL USA; 3 Harvey Cushing/John Hay Whitney Medical Library, Yale University, 333 Cedar Street, New Haven, CT 06520-8014 USA; 4 Department of Environmental Health Sciences, Yale School of Public Health, New Haven, CT USA; 5 Department of Epidemiology of Microbial Diseases, Yale School of Public Health, New Haven, CT USA; 6 University of North Carolina at Chapel Hill Project-China, No. 2 Lujing Road, Guangzhou, 510095 China; 7 School of Medicine, University of North Carolina at Chapel Hill, Chapel Hill, NC USA; 8 Faculty of Infectious and Tropical Diseases, London School of Hygiene and Tropical Medicine, London, UK; 9 Department of Applied Mathematics, University of Waterloo, Waterloo, Ontario Canada; 10 College of Health and Human Sciences, Purdue University, West Lafayette, IN USA; 11 Department of Communications, Boston College, Boston, MA USA; 12 School of Interactive Computing, Georgia Tech, Atlanta, GA USA; 13 Department of Health Policy and Management, Yale School of Public Health, New Haven, CT USA; 14 Center for Information Technology Policy, Princeton University, Princeton, NJ USA

**Keywords:** COVID-19, Misinformation, Health, Online, Mitigate

## Abstract

**Background:**

The duration and impact of the COVID-19 pandemic depends in a large part on individual and societal actions which is influenced by the quality and salience of the information to which they are exposed. Unfortunately, COVID-19 misinformation has proliferated. To date, no systematic efforts have been made to evaluate interventions that mitigate COVID-19-related misinformation. We plan to conduct a scoping review that seeks to fill several of the gaps in the current knowledge of interventions that mitigate COVID-19-related misinformation.

**Methods:**

A scoping review focusing on interventions that mitigate COVID-19 misinformation will be conducted. We will search (from January 2020 onwards) MEDLINE, EMBASE, CINAHL, PsycINFO, Web of Science Core Collection, Africa-Wide Information, Global Health, WHO Global Literature on Coronavirus Disease Database, WHO Global Index Medicus, and Sociological Abstracts. Gray literature will be identified using Disaster Lit, Google Scholar, Open Science Framework, governmental websites, and preprint servers (e.g., EuropePMC, PsyArXiv, MedRxiv, JMIR Preprints). Study selection will conform to Joanna Briggs Institute Reviewers’ Manual 2020 Methodology for JBI Scoping Reviews. Only English language, original studies will be considered for inclusion. Two reviewers will independently screen all citations, full-text articles, and abstract data. A narrative summary of findings will be conducted. Data analysis will involve quantitative (e.g., frequencies) and qualitative (e.g., content and thematic analysis) methods.

**Discussion:**

Original research is urgently needed to design interventions to mitigate COVID-19 misinformation. The planned scoping review will help to address this gap.

**Systematic review registrations:**

Systematic Review Registration: Open Science Framework (osf/io/etw9d).

**Supplementary Information:**

The online version contains supplementary material available at 10.1186/s13643-022-01917-4.

## Background

The COVID-19 pandemic represents a substantial challenge to global human wellbeing. The duration and impact of the pandemic depends in a large part on individual and societal actions and, therefore, to the quality and salience of the information to which people are exposed [1]. Unfortunately, COVID-19 misinformation has proliferated, especially on social media [2]. Misinformation is defined as information that has the features of being false or clearly unsubstantiated, determined based on expert opinion and evidence [3].

COVID-19 misinformation comes in many forms. For example, unlikely theories about the virus being created as a biological weapon in China. As large-scale vaccination has scaled up, COVID-19 vaccine-related misinformation has also appeared, such as false reports about Bill Gates’ plan to equip COVID-19 vaccines with microchips to track and control peoples’ actions [4]. Such misinformation may cause people to turn to ineffective and possibly harmful remedies, overreact (e.g., by hoarding goods) or, more dangerously, underreact (e.g., by engaging in risky behavior and inadvertently spreading the virus). Misinformation may also delay access to care, negatively affecting health outcomes [5]. Such information may also worsen existing fear around vaccines and limit their uptake by a population [6], perhaps reducing the population level of indirect protection from the vaccine, or even preventing herd immunity that could eliminate the virus.

The growing danger of COVID-19-related misinformation has spurred efforts to develop and evaluate interventions designed to mitigate the spread of falsehoods and sustain public trust in evidence-based care. Interventions have employed a variety of strategies designed to debunk misinformation, including accuracy reminders [1], curated infographics [7], and misinformation refutation [8]. While such strategies have shown some potential in reducing the impact of COVID-19 misinformation, individuals tend to mitigate misinformation through other means, most commonly web searches [9]. Thus, despite a growing number of studies that attempt to mitigate COVID-19 misinformation, we are far from knowing when and how to best intervene. In addition, there is a need to capture adverse events related to these types of interventions. Social media interventions also have backfired and increased belief in falsehoods [10].

To date, no systematic efforts have been made to evaluate interventions that mitigate COVID-19-related misinformation. Past reviews have detailed interventions to mitigate misinformation on social media [11–14] and the prevalence of misinformation on social media [15, 16] but have not focused on COVID-19. To provide information that can be used to design effective interventions for COVID-19 misinformation, we plan a scoping review that seeks to compile published evidence in the field to identify gaps in current understanding of experimental evidence regarding the mitigation of COVID-19-related misinformation. We will conduct a scoping review rather than use other methods of research synthesis because scoping reviews are appropriate for mapping an area of research [17]. COVID-19 misinformation intervention research outcomes are likely not sufficiently similar to each other to warrant pooling. The review will take a broad view of COVID-19 misinformation, including aspects related to vaccines, COVID-19 origins, and public health measures.

## Methods/design

The review protocol has been preregistered within the Open Science Framework database (osf/io/etw9d) will be reported in accordance with the reporting guidance provided in the Preferred Reporting Items for Systematic Reviews and Metaanalyses extension for Scoping Reviews (PRISMA-ScR) [18] (see checklist in Additional file 1). Research objectives, inclusion criteria, and methodological techniques will be determined before study commencement using the Joanna Briggs Institute Reviewers’ Manual 2020 Methodology for JBI Scoping Reviews [19]. This process will adhere to the indicated framework: (1) identifying research question; (2) developing comprehensive search strategy; (3) identifying relevant studies; (4) selecting studies; (5) charting data; and (6) collating, summarizing, and reporting results. The study team will develop a search strategy as recommended by the 2020 Methodology for JBI Scoping Reviews.

This scoping review will be conducted by 13 individuals: 12 researchers from several universities worldwide, from a range of disciplines (e.g., public health, medicine, communication studies, mathematics, nursing, computer science, political science), and an informationist from the Harvey Cushing/John Hay Whitney Medical Library at Yale University. As mentioned above, the objective of the scoping review is to develop a better understanding of the current research landscape around interventions to mitigate COVID-19 misinformation by investigating existing studies and gaps in the research. The broad research questions are “what are the benefits and risks of interventions to mitigate COVID-19 misinformation?” and “what are the gaps in the current knowledge base on interventions to mitigate COVID-19 misinformation?” The search strategy will be performed in line with techniques that enhance methodological transparency and improve the reproducibility of the results and evidence synthesis.

### Information sources and search strategy

The primary source of literature will be a structured search of electronic databases (from January 2020 onwards): MEDLINE, EMBASE, CINAHL, PsycINFO, Web of Science Core Collection, Africa-Wide Information, Global Health, WHO Global Literature on Coronavirus Disease Database, WHO Global Index Medicus, and Sociological Abstracts. The secondary source of potentially relevant material will be a search of preprint servers (e.g., EuropePMC, PsyArXiv, MedRxiv, JMIR Preprints), Disaster Lit, Google Scholar (e.g., the first five pages will be searched), Open Science Framework, governmental websites, and the COVID-19 social science research tracker [20]. The references of included documents will be hand-searched to identify any additional evidence sources. We will also conduct forward citation chaining. The search strategy will be designed by a research librarian and peer-reviewed by using the Peer Review of Electronic Search Strategies (PRESS) checklist [21]. A draft search strategy for Scopus is provided in Additional file 2. We will use search terms consistent with our main search to find articles for inclusion. The same keywords for the main search will be used to search gray literature each time. All gray literature will be compiled in a folder and reviewed similarly to articles obtained from our database searches. EndNote, a bibliographic software, will be used to store, organize, and manage all references [22].

### Eligibility criteria

We will include all intervention studies that mitigate COVID-19 misinformation. Only English language studies will be considered for inclusion. Past work suggests that excluding non-English language records from a review has a minimal effect on results [23, 24].

#### Inclusion criteria

Published research (peer-reviewed and gray literature where primary data was collected such as reports, research letters and briefs) investigating interventions that mitigate COVID-19 misinformation (as long as the authors have denoted the topic of study as misinformation) in all populations and settings will be eligible for inclusion.

Only intervention-based studies will be included (e.g., experimental studies, quasi-experimental studies, randomized controlled trials).

There will be no restrictions on the region.

Studies reported only as conference abstracts will also be included, only if we do not have access to the full paper. Conference abstracts are often left out of systematic reviews as they may not contain adequate information to conduct quality assessment or a meta-analysis. Here, we will include conference abstracts as they are often published earlier than full manuscripts [25], which is key to a thorough scoping review on an ongoing phenomenon.

#### Exclusion criteria

Commentaries, correspondences, case reports, case series, editorials, and opinion pieces will be excluded. Case reports and case series often contain relatively limited evidence [26].

Qualitative studies will be excluded.

Non-intervention studies will be excluded.

Governmental, other agency guidelines, and white papers will be excluded. Reviews such as systematic reviews and scoping reviews will be excluded, but we will review the references in these for inclusion, if applicable.

### Screening and selection procedure

All reports identified from the searches will be screened by two reviewers independently. First, titles and abstracts of articles returned from initial searches will be screened based on the eligibility criteria outlined above. Second, full texts will be examined in detail and screened for eligibility. Third, references of all considered articles will be hand-searched to identify any relevant report missed in the search strategy. We will also conduct forward citation chaining. Any disagreements will be resolved by discussion, or if necessary, with a third reviewer. A flow chart showing details of studies included and excluded at each stage of the study selection process will be provided. We will contact authors where necessary if the abstracts do not provide sufficient information [25]. Covidence will be used to manage the title/abstract and full-text screening phases [27].

### Data extraction

Reviewers will undergo a practice exercises till they have a high level of agreement (*>* 0.8 kappa) and then independently extract data from studies. Reviewers will abstract the data using a pretested data extraction template. We will use a standardized coding protocol to collect information such as title of study, authors, date published; study setting; study design; description of methodology; description of study sample; type of intervention; type of misinformation (if any); and main findings. Even though a formal risk of bias is not planned for this scoping review, we will note which studies are pre-prints and have not been formally peer-reviewed. We will also note if some studies fail to report appropriate information.

### Data synthesis

Outcomes and other information collected regarding selected studies will be synthesized using quantitative (e.g., outcomes) and qualitative (e.g., content and thematic analysis) methods, with a narrative summary of findings conducted. Synthesis will be presented in tables, summary data in graphs, and individual data for each study in tables. The broad goal of the synthesis is to identify gaps in research and present recommendations for future research agendas.

## Discussion

There has been limited research that compiles available evidence from various settings regarding interventions that mitigate COVID-19 misinformation. Our review will provide an overview of these studies and synthesize available evidence. We will provide an overview of known gaps in the literature, such as how to target corrective information better and to make it more effective, disrupt the formation of linkages between group identities and false claims, and reduce the flow of cues reinforcing those claims from elites and the media [28]. There is much anecdotal work around COVID-19 misinformation, with few intervention studies. The planned review will highlight areas of research focus and gaps that require more attention and capture adverse events related to these types of interventions. Moreover, the COVID-19 context is evolving, especially with large-scale vaccination in the USA likely affecting misinformation in a rapidly shifting fashion, as well as the scientific evidence around the illness adapting to newer studies being conducted throughout the world. Results will thus provide high-level information to inform, support, and customize the design of interventions to mitigate COVID-19 misinformation. As researchers attempt to minimize COVID-19 misinformation, they need to be aware of scientific evidence to develop interventions to achieve their aim. The planned scoping review seeks to provide this evidence by contributing an evaluation of what is currently known about interventions that mitigate COVID-19 misinformation, with the goal of identifying gaps in research and presenting recommendations for future research foci.

The methodological strength of the planned scoping review is the use of a trans-parent and reproducible procedure for a scoping literature review. We state the data sources, search strategy, and data extraction [29]. Through publishing this research protocol, we strengthen the clarity of the search strategy. The information from the planned review may provide guidance going forward, as COVID-19 likely transforms itself into a seasonal endemic infection like influenza [30]. Thus, some of the insights in the planned review may be helpful in the coming decade. Any amendments to this protocol will be documented in the final published scoping review with reference to saved searches and analysis.

The results of the review will first be posted to a preprint server so that results can be of benefit more quickly and then disseminated in a peer-reviewed journal and likely in other media such as: conferences, seminars, symposia. The protocol and final review article will be made open access upon publication. As per PRISMA-ScR guidelines, we will present results in a user-friendly format [31].

### Limitations

Our planned review should be read in line with some limitations. Although we plan to search several databases and gray literature sources, we may miss some studies; due to the rapid research pace around the COVID-19 pandemic, it is likely that relevant research, crosscutting across different disciplines, exists in fragmented indexing databases. Not all authors we reach out to may respond and we may thus miss some unpublished work. We may not be able to make policy recommendations due to the lack of quality appraisal of studies [32]. The quickly changing COVID-19 context may be a potential limitation.

## Supplementary Information

**Additional file 1.**

**Additional file 2.**

## Data Availability

The datasets used and analyzed will be made available upon reasonable request.

## Abbreviations

PRISMA-ScR: PRISMA Extension for Scoping Reviews JBI: Joanna Briggs Institute
COVID-19: Coronavirus disease 2019
