# Supplementary material for: Interventions to mitigate COVID-19 misinformation: protocol for a scoping review
Source: Syst Rev. 2022 May 30;11:107. doi: 10.1186/s13643-022-01917-4 (PMC9148843; doi:10.1186/s13643-022-01917-4)
Supplement: Supplementary file 2 — Additional file 2. [file 13643_2022_1917_MOESM2_ESM.docx]

# MEDLINE DRAFT SEARCH STRATEGY

**Search strategy**

((TITLE-ABS-KEY(covid* OR sars-ncov-2)) OR (TITLE-ABS-KEY(pandemic) AND PUBYEAR *>* 2019)) AND (TITLE-ABS-KEY(misinformation OR

disinformation OR ”conspiracy theor*” OR literacy OR literate OR correct* OR retract* OR rumor* OR rumour* OR truthful* OR ”fake news” OR fact-check* OR (information W/3 (verified OR unverified OR credib* OR accura* or inaccura* OR trust* or misleading or quality or false)) OR infodemic OR Q OR plandemic OR pseudoscien* OR pseudo-scien* OR conspiratorial OR gossip OR hoax* OR ”urban legend*” OR myth* OR fallac* or falsehood* OR (covid* W/1 deni*))) AND (TITLE-ABS-KEY(correct* OR fact-check* OR refut* OR warn* OR debunk* OR deny OR denial* OR combat* OR educat* OR prophyla* or rebut* or intervention* OR trial* ))
